# Differential changes in bone strength of two inbred mouse strains following administration of a sclerostin-neutralizing antibody during growth

**DOI:** 10.1371/journal.pone.0214520

**Published:** 2019-04-04

**Authors:** Noah J. Mathis, Emily N. Adaniya, Lauren M. Smith, Alexander G. Robling, Karl J. Jepsen, Stephen H. Schlecht

**Affiliations:** 1 School of Medicine, University of Michigan, Ann Arbor, Michigan, United States of America; 2 Department of Anatomy and Cell Biology, Indiana University School of Medicine, Indianapolis, Indiana, United States of America; 3 School of Public Health, University of Michigan, Ann Arbor, Michigan, United States of America; 4 Department of Orthopaedic Surgery, University of Michigan, Ann Arbor, Michigan, United States of America; 5 Department of Mechanical Engineering, University of Michigan, Ann Arbor, Michigan, United States of America; Rensselaer Polytechnic Institute, UNITED STATES

## Abstract

Administration of sclerostin-neutralizing antibody (Scl-Ab) treatment has been shown to elicit an anabolic bone response in growing and adult mice. Prior work characterized the response of individual mouse strains but did not establish whether the impact of Scl-Ab on whole bone strength would vary across different inbred mouse strains. Herein, we tested the hypothesis that two inbred mouse strains (A/J and C57BL/6J (B6)) will show different whole bone strength outcomes following sclerostin-neutralizing antibody (Scl-Ab) treatment during growth (4.5–8.5 weeks of age). Treated B6 femurs showed a significantly greater stiffness (S) (68.8% vs. 46.0%) and maximum load (ML) (84.7% vs. 44.8%) compared to A/J. Although treated A/J and B6 femurs showed greater cortical area (Ct.Ar) similarly relative to their controls (37.7% in A/J and 41.1% in B6), the location of new bone deposition responsible for the greater mass differed between strains and may explain the greater whole bone strength observed in treated B6 mice. A/J femurs showed periosteal expansion and endocortical infilling, while B6 femurs showed periosteal expansion. Post-yield displacement (PYD) was smaller in treated A/J femurs (-61.2%, *p* < 0.001) resulting in greater brittleness compared to controls; an effect not present in B6 mice. Inter-strain differences in S, ML, and PYD led to divergent changes in work-to-fracture (Work). Work was 27.2% (*p* = 0.366) lower in treated A/J mice and 66.2% (*p* < 0.001) greater in treated B6 mice relative to controls. Our data confirmed the anabolic response to Scl-Ab shown by others, and provided evidence suggesting the mechanical benefits of Scl-Ab administration may be modulated by genetic background, with intrinsic growth patterns of these mice guiding the location of new bone deposition. Whether these differential outcomes will persist in adult and elderly mice remains to be determined.

## Introduction

The protein sclerostin, which is a negative regulator of bone formation, inhibits osteoblast differentiation by preventing the binding of *Wnt* ligand to the LRP5/6 receptor [1–4]. Exposure to monoclonal antibodies against sclerostin has been shown to have anabolic effects on bone in rodents, non-human primates, and humans [5–9]. In murine models, the anabolic response of Scl-Ab has been examined in growing and adult inbred rodents [9–12], as well as growing and adult mutant strains [7, 9, 12]. However, the ways in which this anabolic response varies across inbred mouse strains has not been well studied. There is a need to study the ways in which responses to Scl-Ab exposure vary among individuals that are not genetically identical to understand how to better predict outcomes of Scl-Ab administration.

**Fig 1:**
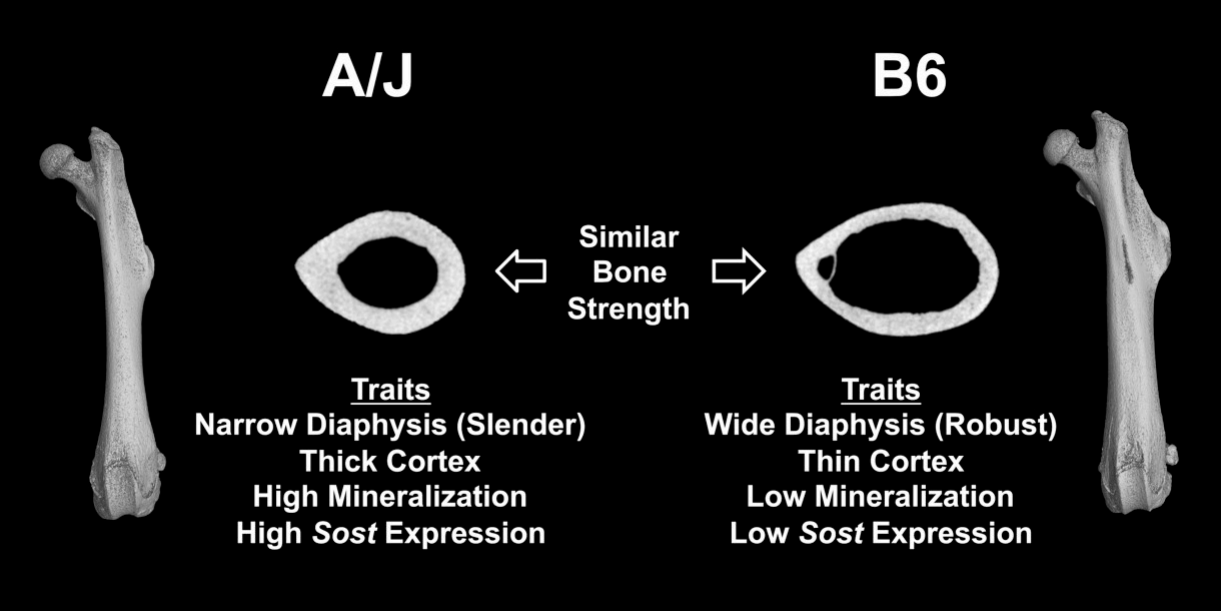
Schematic of key morphological, compositional, and molecular differences between A/J and B6 femurs. Despite variation in traits, both bones establish similar organ level mechanical function at musculoskeletal maturity. Adapted from Schlecht et al [14].

Given that whole bone strength varies with the third power of bone width, engineering principles suggest that baseline bone size may influence treatment outcomes because deposition of new tissue on the outer surface of wide bones would be expected to lead to a greater whole bone strength compared to deposition of the same amount of tissue on the outer surface of narrow bones. Herein, we tested the hypothesis that whole bone strength following administration of Scl-Ab would differ between inbred mouse strains that differ in the external size of their femoral diaphysis and vertebral body. We tested this hypothesis by evaluating the effects of a 4-week treatment of sclerostin-neutralizing antibody on the whole bone strength of long bones and vertebrae of A/J and C57BL/6J (B6) mice. These two strains were chosen not only because they have different external bone sizes, but also because they coordinately adjust morphological and compositional traits similar to that observed for human long bones [13, 14]. A/J femurs are slender (narrow relative to bone length), with a thick cortex, high cortical tissue mineral density (Ct.TMD), and low porosity. In contrast, B6 femurs are robust (wide relative to bone length), with a proportionally thinner cortex, lower Ct.TMD, and higher porosity [14, 15] (Fig 1). The emergent outcome of this coordination is that A/J and B6 long bones show different growth patterns yet achieve similar whole bone mechanical strength at musculoskeletal maturity [14]. Given these differences in external bone size, we tested the hypothesis that the wider B6 long bones would be associated with a greater whole bone strength following administration of Scl-Ab compared to that of the narrow A/J long bones.

## Methods

### Animal husbandry

Male A/J and B6 mice were purchased from The Jackson Laboratory (Bar Harbor, ME, USA) at 3.5 weeks of age. Mice were randomly divided into 4 groups, with Scl-Ab injection groups and sterile dilution buffer groups for both A/J and B6 (n = 14/group). Out of the 14 mice in each group, 5 were used for mRNA analysis and 9 were used for morphological, whole bone mechanical, and histomorphometric analyses. Mice were housed on a 12-hour light/dark cycle with no more than 5 mice per cage and were given water and standard rodent chow *ad libitum*. The timing and duration of treatment were chosen to study perturbations during a critical phase in the development of diaphyseal bone morphology. The Indiana University Purdue University in Indianapolis Institutional Animal Care and Use Committee specifically approved this study. At the completion of the study mice were anesthetized with isoflurane and euthanized via a bilateral pneumothorax.

### Treatment schedule

Mice were allowed one week to acclimate prior to the start of treatment. Beginning at 4.5 weeks of age, the treatment group was given subcutaneous injections of a ratized sclerostin neutralizing antibody (Scl-Ab III; Amgen, Thousand Oaks, CA, USA and UCB, Brussels, Belgium) twice per week for 4 weeks at a dose of 25 mg/kg of body mass. The 25 mg/kg dose of Scl-Ab III is consistent with prior studies and has been shown to increase bone mass in healthy adult rats and mice [5, 12]. Control mice were treated with subcutaneous injections of sterile dilution buffer (vehicle) with an equivalent dose and schedule. The initiation of Scl-Ab injections at 4.5 weeks of age coincided with a transition from early rapid periosteal expansion and cortical bone accrual to the beginning of a phase represented by a steady increase in Tt.Ar and cortical area (Ct.Ar) [14]. This time frame also coincided with up to 6-fold greater *Sost* expression in A/J compared to B6 [13, 14]. At 8.5 weeks of age, body mass (BM) was measured and mice were anesthetized with isoflurane and euthanized. Left femurs were removed and stored in 1x phosphate buffered saline (PBS) solution at -40° C for the biomechanical analysis (n = 9/group). Right femurs were removed from the same mice and stored in 10% neutral buffered formalin (NBF) solution for histomorphometric analysis. The 5^th^ lumbar vertebra was also removed, fixed in 10% NBF, and stored in 70% ethanol for 3D imaging. For the mice that were used for gene expression analysis (n = 5/group), both femurs were removed and immediately snap frozen in liquid nitrogen.

### Baseline and final bone densitometry

Upon arrival, 3.5-week-old mice were anesthetized using isoflurane and scanned in an anterior-posterior orientation using a PIXImus II mouse densitometer (Lunar Corp., Madison, WI, USA) to obtain baseline bone density prior to beginning treatment. When the mice reached 8.5 weeks of age (the end of the treatment period) the mice were anesthetized, rescanned, and euthanized. Bone mineral content (BMC) and area were calculated from the scans for the whole leg (distal to the acetabulum) and spine (L3-L5) regions, and areal bone mineral density (aBMD) was calculated as BMC/bone area.

### Long bone morphology

Whole left femurs were imaged at an 8 μm voxel size using a nano-computed tomography (nanoCT) system (nanotom-s, GE Sensing and Inspection Technologies, GmbH, Wunstorf, Germany). Imaging parameters were set to 90 kV, 375 μA, 1000 ms, 3 averages, and 1 skip, with a 0.3 mm aluminum filter, as described previously [16]. Image volumes were converted to Hounsfield units using a phantom of air, water, and a hydroxyapatite mimicker (1.69 mg/cc; Gammex, Middleton, WI, USA). The region of interest (ROI) was located at the femoral midshaft beginning just distal to the third trochanter and extended 2 mm distally along the diaphysis; this region corresponds with the site of failure following four-point whole bone mechanical loading tests (detailed below). Thresholding was performed for each ROI using Otsu’s method [17]. MicroView v2.2 Advanced Bone Analysis Application software (GE Healthcare Pre-Clinical Imaging, London, ON, Canada) was used to quantify the average total cross-sectional area (Tt.Ar), cortical area (Ct.Ar), marrow area (Ma.Ar), area moments of inertia relative to the bending direction (I_xx_), and cortical tissue mineral density (Ct.TMD) across the ROI. Femur length (Le) was measured from the proximal tip of the greater trochanter to the distal condyles using MicroView.

### Trabecular architecture

Trabecular architecture was quantified at two anatomical sites; the distal femur and the 5^th^ lumbar vertebral body. For the distal femur, ROIs were defined beginning at the distal point of the femoral growth plate and extending proximally for 3.7% of the total femur length. The 5^th^ lumbar vertebral body was scanned on a Scanco μCT-35 micro-computed tomography system using the following conditions: 50 kV, 120 mA, 151 ms integration time, and 10 μm voxel size. Three-dimensional morphometric properties of the cancellous bone were reconstructed and quantified as previously described [18]. For both sites, trabecular architecture parameters included trabecular thickness (Tb.Th), trabecular number (Tb.N) and bone volume fraction (BV/TV).

### Dynamic histomorphometry

All mice were injected with calcein (12 mg/kg, intraperitoneally) at 4 weeks of age, oxytetracycline HCL (80 mg/kg, subcutaneously) at 6 weeks of age, demeocolcyline (40 mg/kg, subcutaneously) at 7.5 weeks of age, and alizarin complexone (20 mg/kg, intraperitoneally) at 8 weeks of age. After euthanasia, the right femur was excised, fixed in 10% NBF, dehydrated in graded ethanols, and embedded undecalcified in polymethylmethacrylate. Midshaft femur sections were cut in the transverse plane (~200 μm thick) using a diamond-embedded wafering saw (Buehler, Inc., Lake Bluff, IL, USA) and ground to a final thickness of ~30 μm. Periosteal and endocortical bone formation parameters were calculated for the first (calcein) and last (alizarin) labels to capture the anabolic response of the entirety of the experiment. The intervening labels (tetracycline and demeclocycline) were visible in the sections but were not used for quantification–they served as backup labels in case the flanking labels were problematic. On each section, the extent of unlabeled perimeter, single-labeled perimeter (sL.Pm), double-labeled perimeter (dL.Pm), and the area between the double labeling were measured with the Osteomeasure quantitation system (Osteometrics, Inc., Decatur, GA, USA). Derived histomorphometric parameters, including the percent mineralizing surface (MS) divided by the bone surface (BS) (MS/BS, %), the mineral apposition rate (MAR, μm/day) and the bone formation rate divided by the bone surface (BFR/BS, μm^3^/μm^2^ per year), were calculated using standard procedures [19].

### Long bone mechanical properties

Whole-bone mechanical properties were measured by loading the left femurs of A/J and B6 mice (n = 9/strain/treatment) to failure in four-point bending at a rate of 0.05 mm/sec (MTS 858 MiniBionix; Eden Prairie, MN, USA), as described previously [14]. Testing was performed at room temperature while bones were kept moist with PBS, and loading occurred with the anterior surface in tension. Mechanical properties calculated from the load-deflection curves included stiffness (S), maximum load (ML), post-yield deflection (PYD), and work-to-fracture (Work), as described previously [20].

### RNA isolation

Gene expression data was obtained from the femoral diaphysis of Scl-Ab and vehicle treated A/J and B6 mice using whole-transcriptome RNA sequencing as well as qPCR. Diaphyses of the left and right femurs were isolated at the time of sacrifice by removing the metaphyses and flushing the marrow. Within 6 minutes of euthanasia, diaphyses were placed in Eppendorf tubes and frozen in liquid nitrogen to preserve RNA integrity. All tissue harvesting and processing was done in an RNase-free environment. RNA was extracted from the diaphyses by pulverizing the right and left femurs in 2 ml of TRIzol (Life Technologies, Grand Island, NY, USA) using a high-speed tissue homogenizer (Model 1000; ThermoFisher Scientific, Waltham, MA, USA) and extracting RNA as previously described [14]. All samples with a 260/280 ratio of at least 1.6 and RNA integrity number of at least 6 were judged to be of sufficient quality for downstream analysis.

### RNA-sequencing methodology

Whole transcriptome RNA sequencing was conducted on the RNA extracts from the paired left and right femurs from 5 mice per strain and treatment. First, each sample was depleted of ribosomal RNA using RiboGone (Clontech, Mountain View, CA, USA), according to the manufacturer’s instructions. An mRNA library was generated for each sample using the SMARTer Stranded RNA-Seq Kit (Clontech, Mountain View, CA, USA). The manufacturer’s protocol was followed precisely for all volumes and settings. mRNA was broken down into segments of about 200 base pairs each, and single stranded cDNA was synthesized from the mRNA. The resulting cDNA fragments were ligated to barcode adapters and purified using Agencourt AMPure XP magnetic beads (Beckman Coulter, Brea, CA, USA). PCR was run to amplify the cDNA and create RNA- sequencing libraries using DNA polymerase, a universal forward primer, and reverse primers corresponding to the paired-end Illumina primer index. The amplified library was purified again using Agencourt AMPure XP magnetic beads and sample quality was verified with an Agilent Technologies Model 2100 bioanalyzer (Agilent Technologies, Santa Clara, CA, USA). Equal volumes of stranded cDNA libraries for each sample (n = 5/treatment/strain) were pooled together. Each resulting pool (n = 1/treatment/strain) was sequenced in quadruplicate using a HiSeq 2500 System (Illumina, San Diego, CA, USA) to obtain a minimum of 25 million base pair reads per library for statistical analyses between the four pooled samples.

The quality of the raw RNA-sequencing reads was checked using FastQC v1.10.1 (www.bioinformatics.bbsrc.ac.uk/projects/fastqc). Reads were aligned to the reference genome for their respective strain using TopHat v2.0.9 and Bowtie v2.1.0. The reference genomes were obtained from the Center of Genome Dynamics at the Jackson Laboratory (http://cgd.jax.org/tools/Seqnature.shtml). Alignment was checked using FastQC to ensure accurate alignments were used to quantify gene expression differences. Expression of genes identified was quantified using the htseq-count embedded in HTseq [21]. Only reads that mapped unambiguously to a single gene were used, and those that aligned with multiple positions or overlapped with multiple genes were discarded. Gene-level differential expression analyses were conducted using edgeR [22]. Because of the high number of genes in the analysis, *p*-values were adjusted for multiple hypotheses testing using a Benjamini-Hochberg procedure (*q* = 0.05) to prevent false discoveries. Therefore, for a gene to be considered differentially expressed, it needed to have a *q*-value less than or equal to 0.05 as well as a fold change greater than or equal to 1.5. Data was presented as a pathway enrichment analysis using Gene Ontology [23]. Pathways and functional groups that were significantly differentially expressed were found using iPathwayGuide software (Advaita Bioinformatics, Plymouth, MI, USA). Statistical analysis consisted of a Fisher’s exact test, and again a Benjamini-Hochberg correction was used to account for multiple comparisons (*q* = 0.05).

### qPCR analysis

Quantitative PCR was used to confirm and validate results from RNA-sequencing. RNA extracts from the left and right femurs of two mice per treatment and strain were pooled (200 ng of RNA per pool), resulting in four individual pools (i.e., 1 pool for each treatment and strain group) for gene expression analyses. To find differentially expressed *Wnt*-associated genes, TaqMan microfluidic array cards (Applied Biosystems, Foster City, CA, USA) were used which probed for 46 *Wnt* pathway-associated genes and two endogenous controls. cDNA was generated for each pool with qScript cDNA SuperMix (Quanta Biosciences, Gaithersburg, MD, USA) and incubation in a C1000 Thermal Cycler (Bio-Rad Laboratories, Hercules, CA, USA) according to the manufacturer’s protocol. Each resulting cDNA sample was mixed in a 1:1 ratio with TaqMan Gene Expression Master Mix (Applied Biosystems, Foster City, CA, USA) and placed into a custom 384-well microfluidic array card, which used 46 PCR primers specific for genes in the canonical and non-canonical *Wnt* pathways as well as two endogenous control genes. cDNA samples from each of the 4 pools were run simultaneously on the same card in duplicate. The card was centrifuged at 4° C (Legend XTR (with custom TaqMan array card bucket), Sorvall, Waltham, MA, USA). qPCR was conducted in accordance with the manufacturer’s protocol.

Following qPCR data collection, the baseline threshold settings were adjusted to obtain a threshold cycle (CT) that was constant across both strains. The delta-delta CT method was used to calculate fold changes in gene expression, using the beta-2-microglobulin gene as an endogenous reference [24] Genes with fold differences of 1.5 or greater that were shown to be significantly different using a Student’s t-test (*p* < 0.05) were deemed to be differentially expressed.

### Statistical analysis

Data were analyzed using Prism v6 (GraphPad Software, La Jolla, CA, USA) and Minitab v16 (State College, PA, USA). Normal distribution of the data was tested using a Shapiro-Wilk test. Body mass was 11% different between A/J and B6 control mice and between A/J and B6 treated mice. To adequately adjust the data for body mass, a linear regression was used to derive body mass adjusted traits for A/J and B6 mice separately prior to further statistical analyses. Linear regression adjusted trait values were entered into a two-way analysis of variance (ANOVA) to test for strain and treatment main effects, and strain by treatment interactions with a significance level of *p* < 0.05. Tukey multiple comparisons test between Scl-Ab and vehicle treated mice within and between strains were assessed to determine the significance of least square mean differences in the presence of important interactions. These mean values were used to calculate percent differences between groups as presented in the following figures and text.

## Results

The means of the unadjusted morphological traits and mechanical properties are reported in Table 1, along with body mass data. All raw data is freely available within the Dryad Digital Repository (doi:10.5061/dryad.qp321v7).

**Table 1:** Final body mass at study completion with unadjusted means and standard deviations for morphological and mechanical traits of Scl-Ab and vehicle treated femurs from A/J and B6 inbred mice. Significant differences prior to body mass adjustment (*p* < 0.05) between vehicle and treated mice within a strain are indicated in **bold**.

**Strain**	**Tx**	**BM** **(g)**	**Ct.Ar** **(mm**^**2**^**)**	**Tt.Ar** **(mm**^**2**^**)**	**Ma.Ar** **(mm**^**2**^**)**	**Ct.TMD** **(mg/cc)**	**S** **(N/mm)**	**ML** **(N)**	**PYD** **(mm)**	**Work** **(Nmm)**
**A/J**	Veh	19.75 ±1.95	0.59 ±0.03	1.12 ±0.06	0.54 ±0.04	1338 ±37	112.3 ±19.0	18.3 ±1.4	0.39 ±0.09	7.36 ±1.51
	Scl-Ab	19.08 ±1.13	**0.79** **±0.05**	**1.25** **±0.08**	**0.46** **±0.04**	1336 ±39	**154.8** **±31.1**	**24.6** **±4.7**	**0.14** **±0.03**	**4.81** **±2.01**
**B6**	Veh	22.10 ±2.01	0.77 ±0.11	1.77 ±0.21	1.00 ±0.11	1264 ±45	111.1 ±30.8	19.3 ±3.4	0.59 ±0.11	10.76 ±3.62
	Scl-Ab	21.51 ±1.59	**1.04** **±0.14**	**1.99** **±0.19**	0.95 ±0.07	1256 ±43	**176.5** **±25.2**	**33.7** **±4.4**	0.50 ±0.11	**16.42** **±4.22**

### Differential effect of Scl-Ab on whole bone mechanical properties

**Fig 2:**
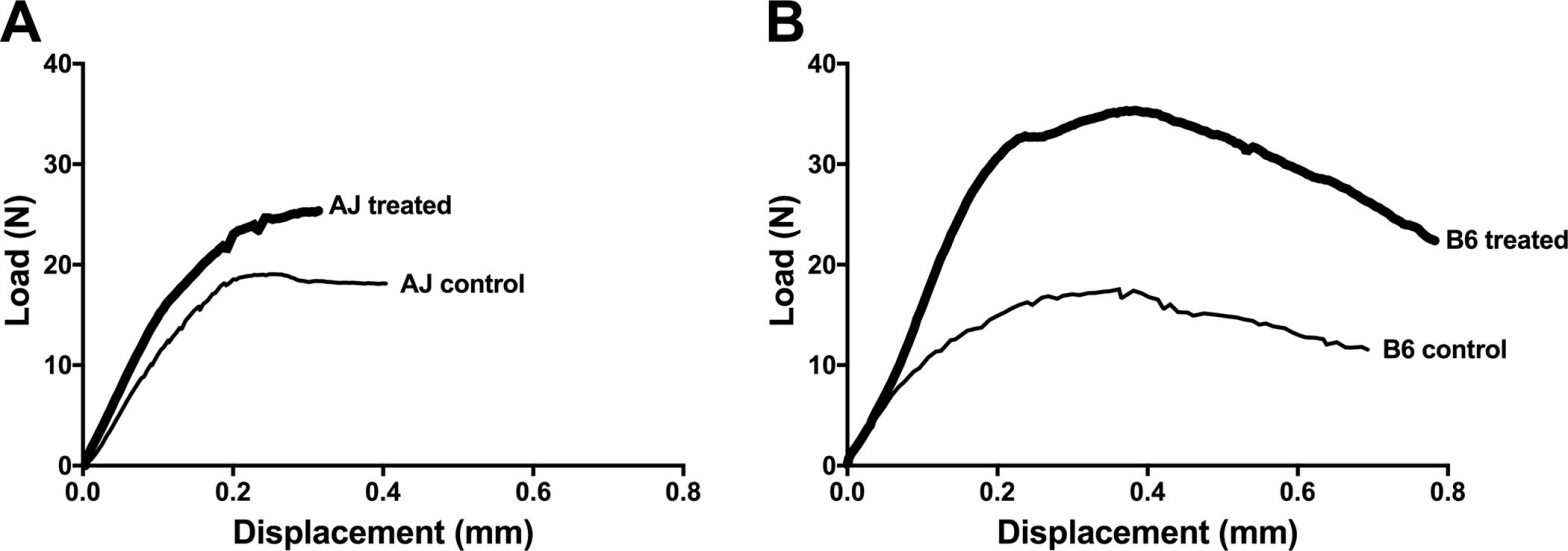
Representative load-deflection curves from four-point bending. A) A/J Veh and A/J Scl-Ab, and B) B6 Veh and B6 Scl-Ab. Stiffness was calculated as the slope of the linear portion of the curve, maximum (max) load was calculated as the highest point on the curve, PYD was the displacement between the end of the linear portion of the curve and the point of failure, and work-to-fracture was calculated as area under the curve put to the point of failure.

**Fig 3:**
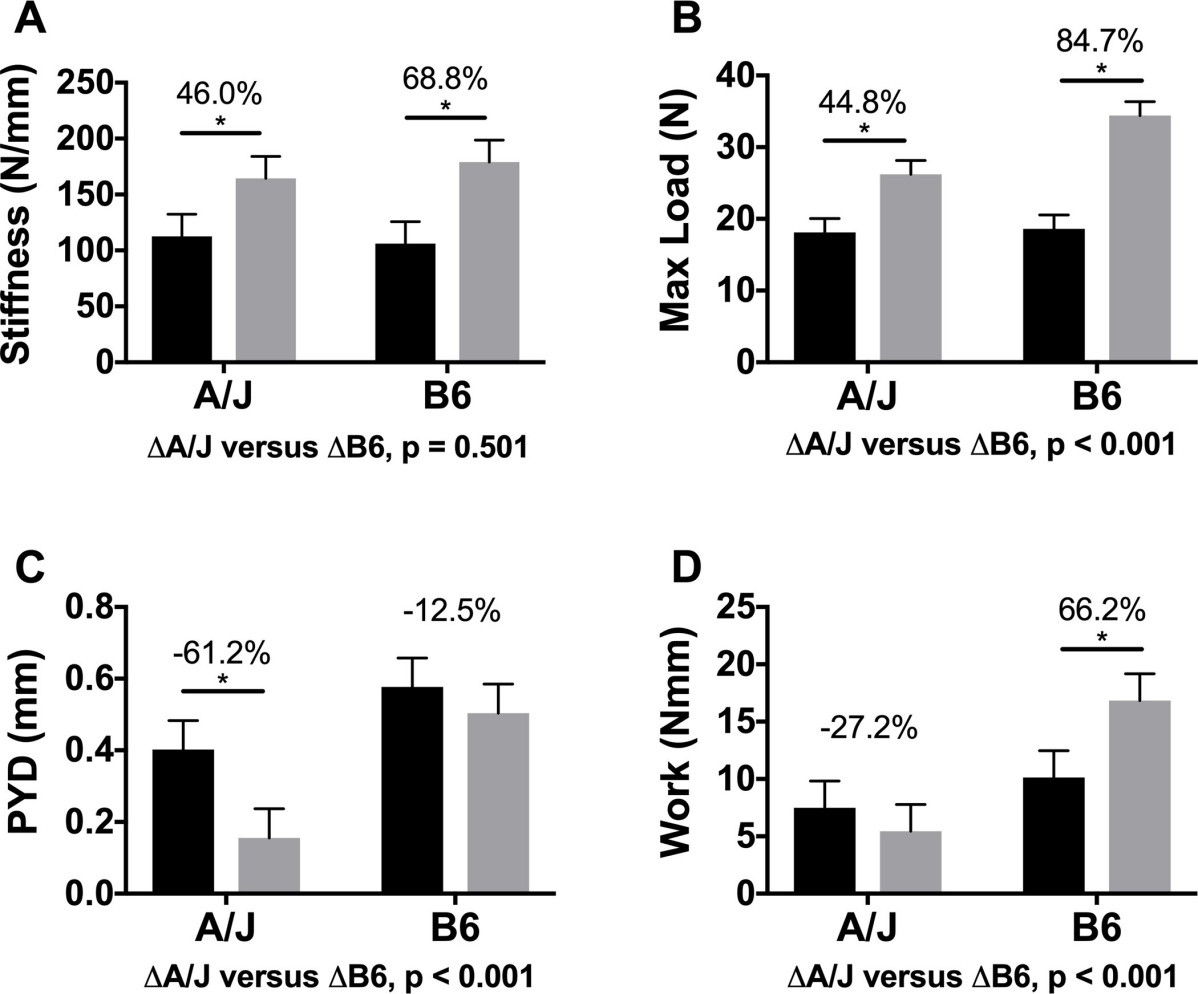
Four-point bending of the femurs of treated and untreated mice. Both strains of treated mice showed greater whole bone A) stiffness and B) strength compared to their respective controls. C) PYD was significantly smaller with treatment in A/J but not B6, and D) Work showed divergent outcomes, being significantly smaller in treated A/J femurs but significantly greater in treated B6 femurs compared to their respective controls. Main effects by strain and treatment as well as the interaction of strain by treatment were tested using an ANOVA. Percent changes were calculated from least square mean differences of Tukey pairwise comparisons (*p* < 0.05). Black bars = Vehicle and gray bars = Scl-Ab treated.

All mechanical properties were calculated from load-deflection curves generated by 4-point bending. Representative load-deflection curves are provided in Fig 2. Treated A/J femurs showed a 46.0% (*p* < 0.001) greater S (Fig 3A) and 44.8% (*p* < 0.001) greater ML (Fig 3B) relative to controls. In contrast, treated B6 femurs showed a 68.8% (*p* < 0.001) greater S (Fig 3A) and 84.7% (*p* < 0.001) greater ML (Fig 3B) relative to controls. Scl-Ab exposure resulted in a greater ML (*p* < 0.001) in B6 femurs compared to that of A/J, but there was no difference in S (*p* = 0.501). The strain by treatment interaction was significant for ML (F = 29.56, *p* < 0.001), but not for S (F = 2.19, *p* = 0.150).

PYD, a measure of brittleness, also showed differential effects with treatment between strains as illustrated by the significant strain by treatment interaction (F = 8.64, *p* = 0.007). Femurs from treated A/J mice showed a significant 61.2% smaller PYD relative to controls (*p* < 0.001). In contrast, the femurs of treated B6 mice showed a non-significant 12.5% smaller PYD relative to controls (*p* = 0.312) (Fig 3C). The smaller PYD shown in treated A/J femurs relative to their controls was significantly different from the smaller PYD for treated B6 femurs (*p* < 0.001) compared to their controls. The changes in Work between treated and untreated groups differed between A/J and B6 femurs. Femurs from Scl-Ab treated A/J mice showed a 27.2% smaller Work compared to the control A/J group (*p* = 0.366). In contrast, B6 mice treated with Scl-Ab exhibited a significant 66.2% greater Work compared to controls (*p* < 0.001) (Fig 3D). These responses to Scl-Ab were significantly different between A/J and B6 (*p* < 0.001). The strain by treatment interaction was also significant (F = 26.39, *p* < 0.001).

### Changes in long bone morphology and cortical tissue-mineral density

**Fig 4:**
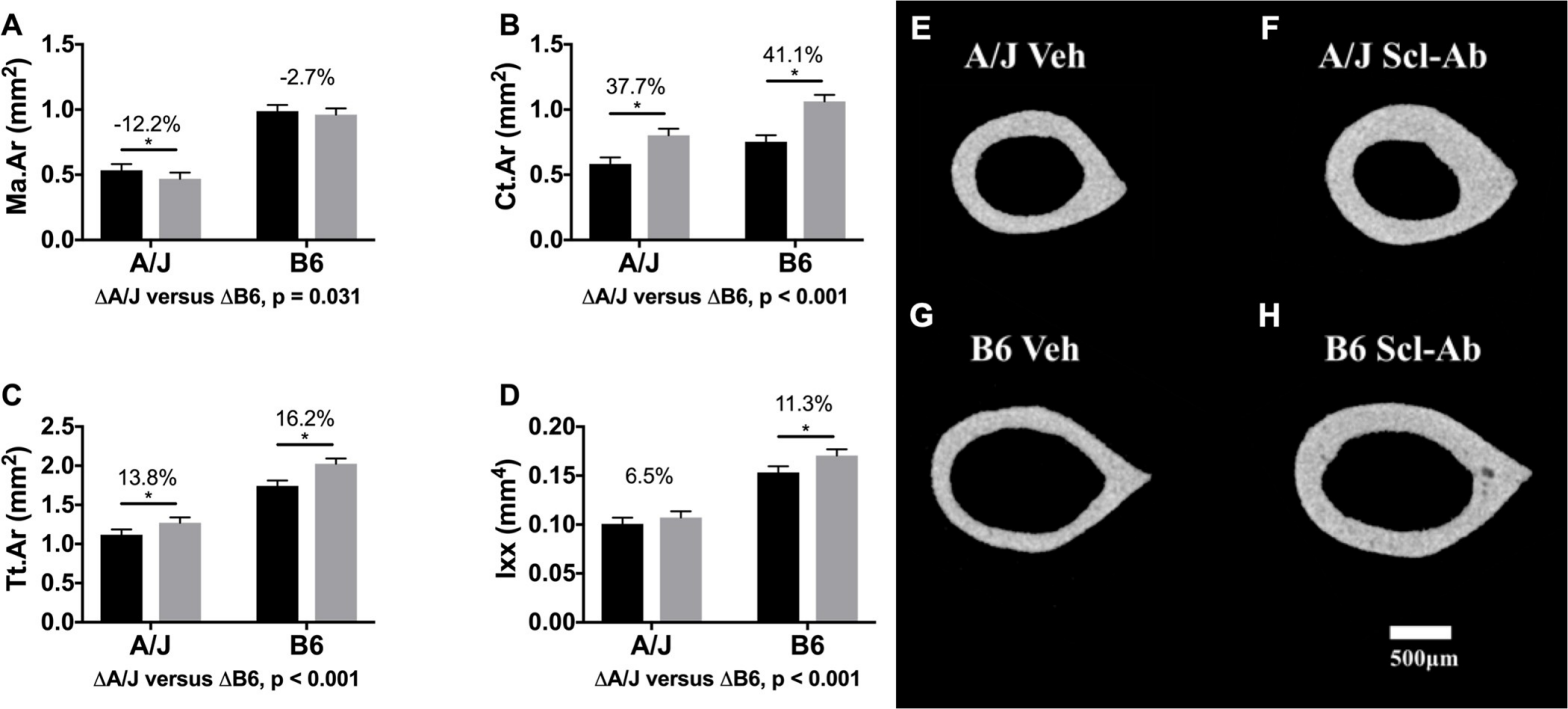
Morphological data derived from the femoral diaphysis. Data showed that Ct.Ar (A) and Tt.Ar (B) were similarly significantly greater in A/J and B6 femurs, while Ma.Ar (C) was significantly smaller only in A/J femurs with treatment. Moment of inertia (D) was significantly greater with Scl-Ab administration in both strains, but the magnitude of this change was significantly greater in B6 than A/J. Main effects by strain and treatment as well as the interaction of strain by treatment were tested using an ANOVA. Percent changes were calculated from least square mean differences of Tukey pairwise comparisons (p < 0.05). Representative nanoCT images of mid-diaphyseal cortical bone for treated and control A/J and B6 mice are shown in Fig 4E–4H. Black bars = Vehicle and gray bars = Scl-Ab treated.

Cross-sectional morphology of the femoral midshaft was assessed to test if A/J and B6 femurs responded differently to Scl-Ab exposure. Unadjusted morphological traits are listed in Table 1. After adjusting for body mass differences between strains, Scl-Ab treated A/J mice showed a 37.7% greater femoral Ct.Ar (*p* < 0.001) relative to control A/J femurs (Fig 4A). This relatively greater cortical area in A/J resulted from a 13.8% greater Tt.Ar (*p* < 0.001) and a 12.2% smaller Ma.Ar (*p* = 0.031) relative to untreated A/J mice (Fig 4B and 4C). Treated B6 mice also showed a similar 41.1% greater Ct.Ar relative to untreated B6 mice (*p* < 0.001) (Fig 4A). The greater Ct.Ar of treated B6 mice resulted primarily from a 16.2% greater Tt.Ar (*p* < 0.001) and only a 2.7% smaller Ma.Ar (*p* = 0.644) (Fig 4B and 4C). Neither treated A/J nor treated B6 femurs showed a change in Ct.TMD relative to controls (-0.1% in A/J, *p* = 0.999; -1.3% in B6, *p* = 0.798) (Table 2). The changes in Ct.Ar (*p* < 0.001), Tt.Ar (*p* < 0.001) and Ma.Ar (*p* < 0.001) were significantly different when comparing the least squares means of treated A/J and B6 mice. The strain by treatment interaction was significant for Ct.Ar (F = 7.24, *p* = 0.011) and Tt.Ar (F = 7.95, p = 0.008), but not Ma.Ar (F = 1.5, *p* = 0.229). This indicates a differential response by strain for Ct.Ar and Tt.Ar with treatment but not for Ma.Ar. Overall, the changes in femoral morphology of treated A/J and B6 femurs were reflected in the DXA results with both showing a significantly greater whole leg aBMD. However, DXA was unable to detect the differential distribution of new bone between mouse strains (Table 2).

**Table 2:** Areal bone mineral density (aBMD) data from PIXImus scans of the whole leg and spine of A/J and B6 mice treated with Scl-Ab or vehicle. Scans were conducted prior to the start of dosing (4 weeks) and following the completion of dosing (8 weeks).

Strain	Whole Leg		Spine	
	Wk. 4	Wk. 8	Wk. 4	Wk.8
**A/J**				
**Vehicle**	0.0357 ±0.0020	0.0447 ±0.0015	0.0368 ±0.0010	0.0511 ±0.0055
**Scl-Ab**	0.0369 ±0.0007	0.0542* ±0.0016	0.0371 ±0.0023	0.0750* ±0.0047
**B6**				
**Vehicle**	0.0349 ±0.0018	0.0478 ±0.0046	0.0379 ±0.0043	0.0531 ±0.0063
**Scl-Ab**	0.0350 ±0.0014	0.0591* ±0.0046	0.0348 ±0.0011	0.0789* ±0.0050

* denotes a significant difference between vehicle and Scl-Ab treated groups within strains (*p* < 0.05). There were no significant differences in responses between strains.

### Changes in trabecular architecture

**Fig 5:**
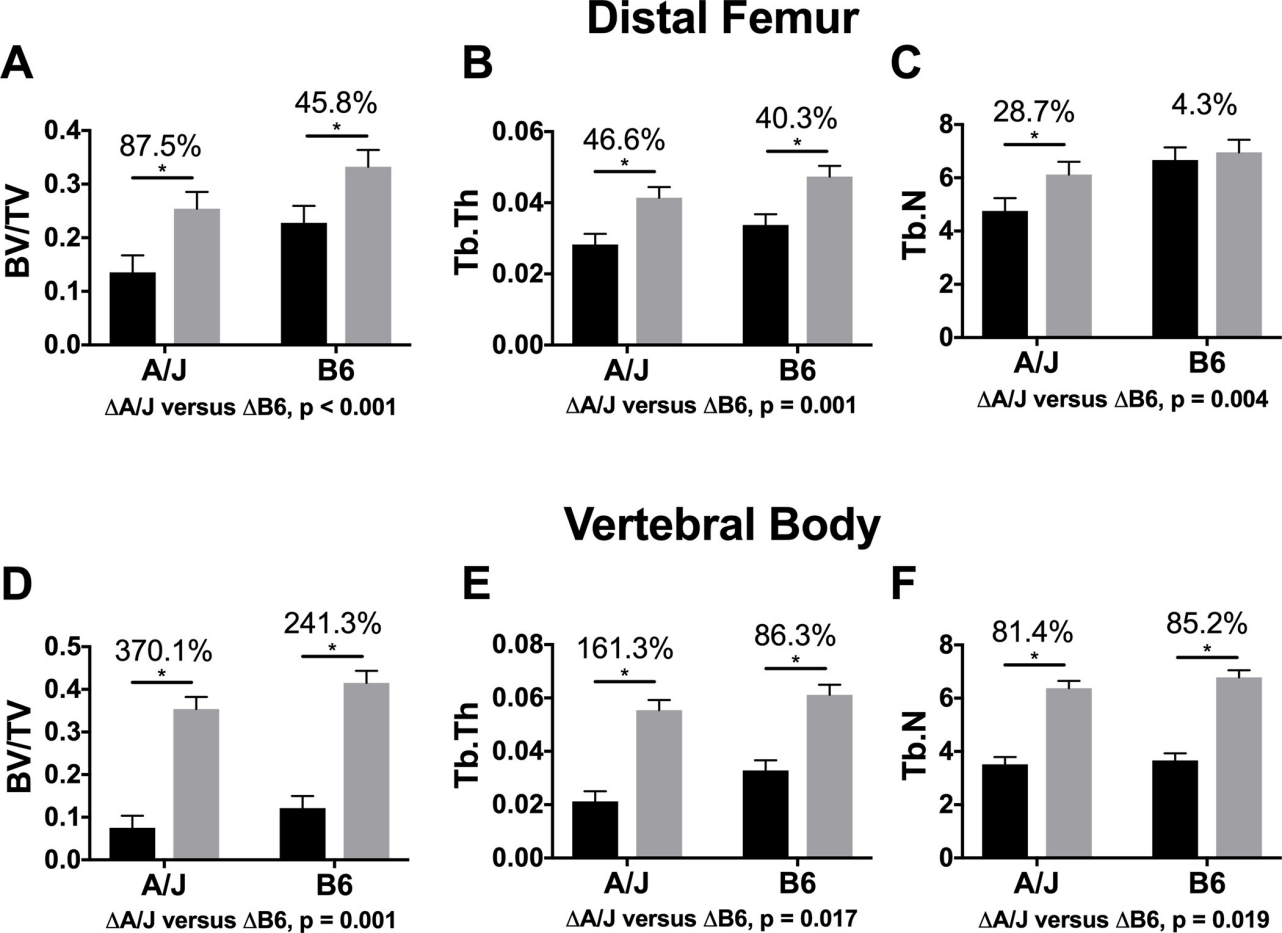
Analysis of nanoCT images acquired for the distal femur. Data showed a greater bone volume fraction (A) in treated A/J and B6 femurs compared to controls. This was a result of a significantly greater trabecular number for treated A/J but not for B6 (B), and a significantly greater trabecular thickness for both treated A/J and B6 femurs (C). Analysis of nanoCT images acquired for the vertebral body revealed significantly greater trabecular bone volume fraction (D) for both A/J and B6, resulting from both greater Tb.Th (E) and Tb.N (F) with treatment. Main effects by strain and treatment as well as the interaction of strain by treatment were tested using an ANOVA. Percent changes were calculated from least square mean differences of Tukey pairwise comparisons (p < 0.05). Black bars = Vehicle and gray bars = Scl-Ab treated.

Analysis of trabecular architecture in the distal femur of A/J mice revealed a significant 87.5% greater BV/TV (*p* < 0.001) in Scl-Ab treated mice relative to control A/J mice (Fig 5A). This greater BV/TV resulted from a significant 46.6% greater Tb.Th (*p* < 0.001) and a 28.7% greater Tb.N (*p* < 0.001) in treated A/J mice relative to the controls (Fig 5B and 5C). In B6 mice, the treated group demonstrated a 45.8% greater BV/TV (*p* < 0.001) (Fig 5A). The greater BV/TV with Scl-Ab treatment in B6 mice were a result of a significant 40.3% greater Tb.Th (*p* < 0.001) but no significant change in Tb.N (4.3%, *p* = 0.588) (Fig 5B and 5C). Changes between A/J and B6 with treatment were significantly different in BV/TV (*p* < 0.001), Tb.N (*p* = 0.004), and Tb.Th (*p* = 0.001). The strain by treatment interaction was significant for distal femora Tb.N (F = 11.59, *p* = 0.002), but not for either BV/TV (F = 0.45, *p* = 0.507) or Tb.Th (F = 0.05, *p* = 0.826). Similarly, in the vertebral body, there was greater BV/TV in treated A/J and B6 mice relative to their controls (*p* < 0.001) (Fig 5D). Tb.Th was also significantly greater in both A/J and B6 with Scl-Ab treatment, (*p* < 0.001) (Fig 5E), as was Tb.N. (p < 0.001) (Fig 5F). Vertebral BV/TV, Tb.N, and Tb.Th was significantly different between treated A/J and B6 mice (*p* < 0.001, *p* = 0.019, and *p* = 0.017, respectively). The strain by treatment interaction was significant for vertebral Tb.Th (F = 5.02, *p* = 0.033), but not for either BV/TV (F = 0.59, *p* = 0.449) or Tb.N (F = 1.90, *p* = 0.179) The changes in trabecular architecture of treated A/J and B6 femurs were reflected in their significantly greater spine aBMD (Table 2).

### Dynamic histomorphometry

**Fig 6:**
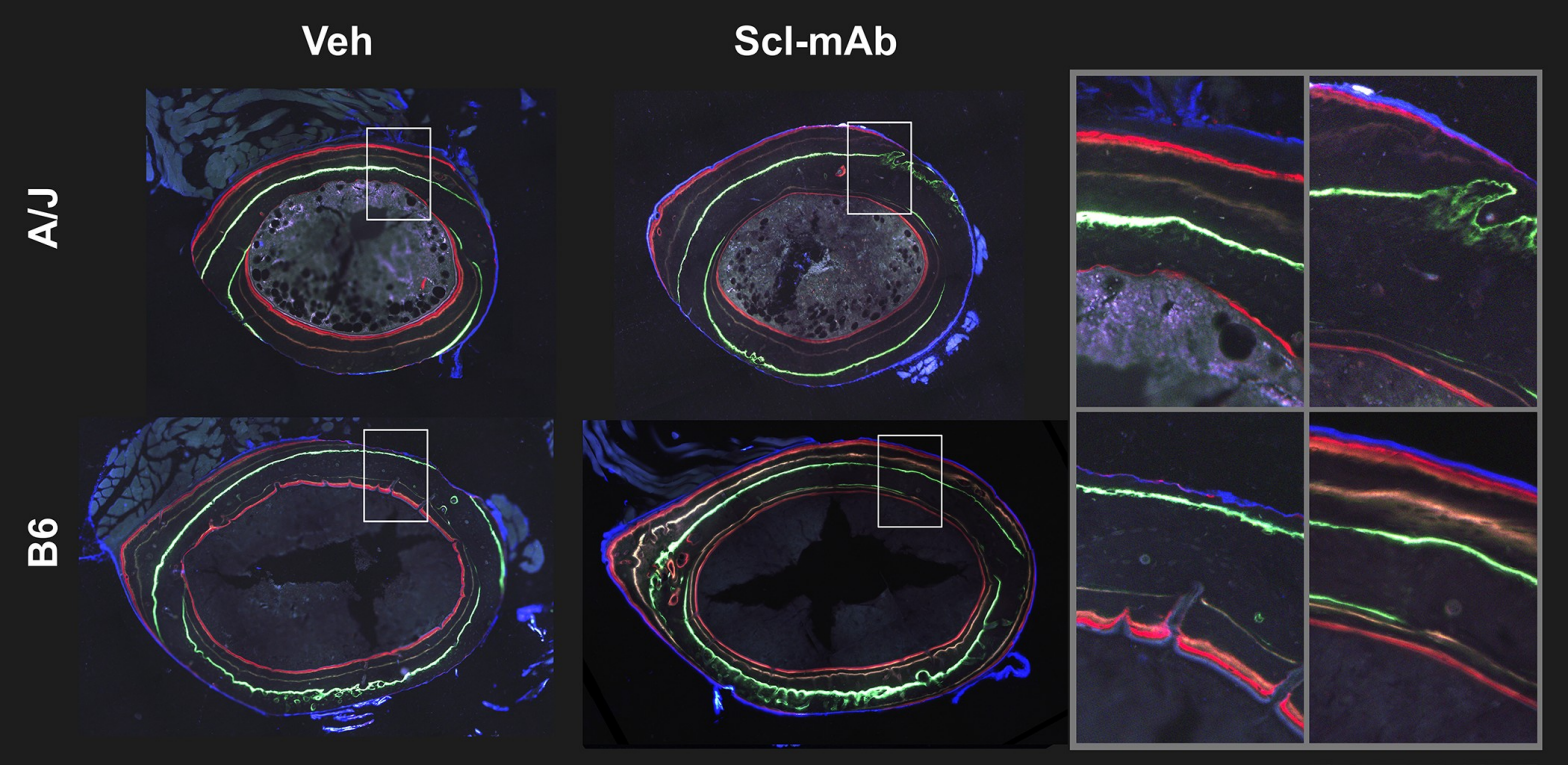
Representative histological images of the 4 fluorescent bone labels for treated and untreated A/J and B6 mice. Calcein was administered at 4 weeks of age, oxytetracycline HCL was administered at 6 weeks of age, demeocolcyline was administered at 7.5 weeks of age, and alizarin complexone was administered at 8 weeks of age.

**Fig 7:**
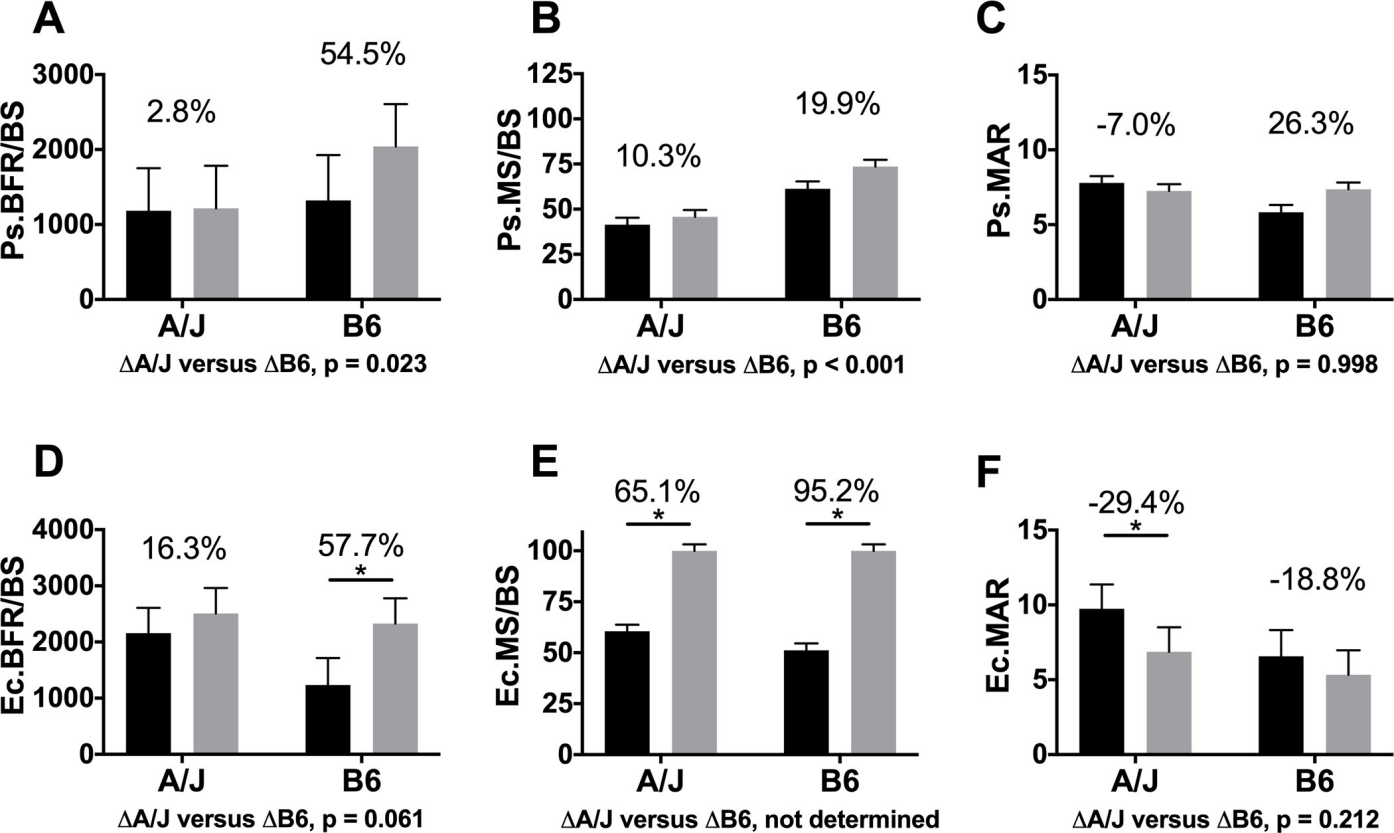
Dynamic histomorphometry analysis. Data revealed a trend towards higher periosteal bone formation rate per unit of bone surface (BFR/BS) in B6 femurs, but not A/J (A), as well as a significantly greater Ec.BFR/BS in B6 femurs, and a trend towards higher Ec.BFR/BS in A/J (D). These changes resulted from greater MS/BS with Scl-Ab treatment on periosteal (Ps.) (B) and endocortical (Ec.) (E) surfaces in A/J and B6 mid-femoral cortex, along with changes in mineral apposition rate (MAR) (C & F). Main effects by strain and treatment as well as the interaction of strain by treatment were tested using an ANOVA. Percent changes were calculated from least square mean differences of Tukey pairwise comparisons (p < 0.05). Black bars = Vehicle and gray bars = Scl-Ab treated.

New tissue was added primarily along the posterior-anterior axial plane (Fig 6). In A/J mice, BFR/BS on the periosteal surface (Ps.BFR/BS) was not different in Scl-Ab and untreated groups (2.8%, *p* = 0.999) (Fig 7A). However, in B6 mice, Scl-Ab treatment led to a trend towards higher Ps.BFR/BS (54.5%, *p* = 0.067). On the endocortical surface, Scl-Ab treatment led to a significant effect on BFR/BS (Ec.BFR/BS) in B6 mice (57.7%, *p* = 0.016), but not in A/J femurs (16.3%, *p* = 0.367) (Fig 7D). Notably, the baseline Ec.BFR/BS in control mice was significantly higher in A/J than B6 (74.8%, *p* = 0.001), indicating that at 8 weeks of age, A/J control mice showed a greater baseline degree of endocortical formation than B6 controls, even though B6 showed a stronger response to Scl-Ab treatment. Finally, each mouse in both A/J Scl-Ab and B6 Scl-Ab groups showed 100% MS/BS, which prevented us from calculating a *p*-value comparing the least square mean difference between A/J and B6 because there was no distribution of trait values for A/J Scl-Ab or B6 Scl-Ab groups. The strain by treatment interaction was trending towards significance for Ps.BFR/BS (F = 3.19, *p* = 0.085) and was not significant for Ec.BFR/BS (F = 1.38, *p* = 0.251).

### Few genes were differentially expressed between Scl-Ab treated and vehicle controls

RNA sequencing was used to detect differences in expression of individual genes and pathways after the 4-week study between A/J control and B6 control (S1 Table), between A/J Scl-Ab and A/J control (S2 Table), and between B6 Scl-Ab and B6 control (S3 Table). Gene expression profile data is freely available within the Dryad Digital Repository (doi:10.5061/dryad.qp321v7). In comparing A/J versus B6 controls, we showed 4084 differentially expressed genes and several enriched pathways, including the canonical *Wnt* signaling pathway. This was consistent with gene expression differences in 8 week old A/J and B6 mice reported previously [14]. Comparing gene expression profiles of femurs from treated A/J mice to A/J control mice in this study, 463 genes out of 19,917 genes with measured expression were found to be differentially expressed. The differentially expressed genes were primarily from pathways involved in the development and function of skeletal and cardiac muscle. Two genes known to be involved in the *Wnt* signaling pathway (*Camk2b*, *Fzd1*) were differentially expressed between A/J Scl-Ab and A/J controls. Despite the differences observed in bone morphological and mechanical properties between antibody and vehicle treated mice, no pathways known to be involved in bone development or function were enriched in femurs from treated A/J mice relative to controls. Further, a qPCR array of *Wnt* pathway genes did not show large differences between A/J Scl-Ab and A/J controls. The only differentially expressed *Wnt* pathway gene revealed by qPCR was *Msx2*.

The comparison between treated and control B6 femurs revealed no differentially expressed genes and no enriched pathways. The lack of a large gene expression level response to treatment was substantiated by qPCR data, which showed only one differentially expressed gene, *Sfrp4*, which was upregulated in femurs from Scl-Ab treated B6 mice relative to vehicle treatment.

## Discussion

Our results supported the hypothesis that treatment with Scl-Ab is associated with a significantly greater whole bone stiffness and strength for the wide bones of B6 mice compared to the narrow bones of A/J mice. This outcome is consistent with engineering principles. Importantly, the greater strength benefit of Scl-Ab treatment was reflected in Work to failure for only the B6 femurs. A/J femurs showed no significant change in Work, whereas B6 femurs showed a significantly greater Work. The decision to administer Scl-Ab beginning at 4 weeks of age was based on testing our hypothesis during a rapid growth phase, exacerbating intrinsic differences in bone growth patterns known for A/J and B6 mice [13, 25]. Ending this study during adolescence, when A/J and B6 mice are still growing is a limitation. However, the purpose of our study was to examine whether there was a differential effect on whole bone strength after administering Scl-Ab in a model that mirrors the variation within bone morphology observed during growth [26, 27] and upon adulthood among humans [28–30]. This study was not intended to introduce a pre-clinical model to assess pharmacologic treatment outcomes.

B6 femurs showed a significantly greater whole bone strength relative to their untreated controls compared to A/J femurs, as expected. Given that both strains showed relatively similar changes in cortical area with Scl-Ab treatment, the greater mechanical benefit observed for B6 mice appears to have resulted in part from differences in baseline morphology combined with minor differences in the spatial deposition of new tissue with treatment. The additional tissue deposited following Scl-Ab administration was located primarily along the posterior-anterior direction (Fig 6), which is consistent with the normal cortical drift pattern associated with mouse femoral diaphyseal growth [31]. This bone deposition location is perpendicular to the plane of loading and thus amenable to being readily observed with standard four-point bending failure tests. Because B6 femurs have a larger external size than A/J, the newly deposited cortical matrix in treated B6 femurs was positioned further from the bending plane (geometric centroid) thereby resulting in a greater mechanical benefit of Scl-Ab treatment compared to A/J femurs, as expected. Treated A/J femurs showed significantly smaller changes in Ma.Ar relative to changes in treated B6 femurs (-12.2% in A/J, -2.7% in B6). This suggested that the new bone deposition associated with Scl-Ab treatment in A/J femurs was distributed along both the endocortical and periosteal surfaces, whereas in B6 femurs new tissue was deposited primarily along the periosteal surface, consistent with work by others [9, 12, 32]. Thus, both A/J and B6 mice showed greater whole bone strength and cortical volume following Scl-Ab treatment; however, spatial differences in where new bone was deposited also contributed to the greater mechanical benefit in treated B6 long bones compared to A/J. These results are consistent with studies of Scl-Ab in growing mice as a way to rescue whole bone strength in a mouse model of osteogenesis imperfecta (OI) [9, 12]. Human OI bone is known to have lower cortical mass arising from a more slender bone phenotype [33] and our results may help explain why the treated OI mutant mice may have showed only a 51% increase in whole bone strength compared to the 68% increase observed for the control mice [9]. Our results and the outcomes shown by others confirm the potent anabolic effect of Scl-Ab [5], yet also show there are nuances in the response to treatment that may depend on genetic background. Whether these differences in whole bone strength outcomes will occur for adult or elderly A/J and B6 mice remains to be determined.

A surprising outcome of the four-point bending tests was that A/J femurs were more brittle relative to vehicle controls following Scl-Ab treatment. During normal development, A/J mice have a naturally lower PYD compared to B6 mice, arising from the coordinately increased mineral content, as measured by ash content, that mechanically offsets the smaller external bone width but makes A/J long bones more brittle [20]. Treating A/J mice with Scl-Ab further lowered PYD, an effect not observed in B6 femurs. The differences in PYD following Scl-Ab treatment explained the divergent changes in Work for the two mouse strains. Because Work is measured as the area under the load-deflection curve, changes in Work depend on changes in S, ML, and PYD. The smaller PYD observed in treated A/J femurs was sufficient in magnitude to offset the greater S and ML leading to no change in Work. In B6, the non-significant smaller PYD as well as the greater ML led to the B6 femurs exhibiting greater Work. This divergent outcome was unexpected since it is in contrast with prior studies showing greater Work with Scl-Ab treatment [5, 12]. However, finding that the brittleness of A/J femurs was not improved following Scl-Ab treatment is consistent with the work of others [9, 12] which showed Brtl/+ mice (Type IV osteogenesis imperfecta (OI) model) treated with a sclerostin antibody failed to significantly improve tissue ductility. Our findings in A/J femurs, along with these OI studies, suggest that sequestration of sclerostin in animals with a brittle phenotype can improve whole bone strength but may not address the brittle nature of the cortical matrix. Though the goal of this study was not to assess the utility of Scl-Ab for treatment of disease during growth, the observation of divergent outcomes that depend on an individual’s genetic background could be of clinical value. A limitation of this study was a lack of information on tissue-level mechanical properties, which may provide additional insight into the material response of bone to Scl-Ab treatment, in particular the material changes contributing to the smaller PYD of A/J femurs.

Our previous work showed that A/J and B6 mice differ in femoral morphology, Ct.TMD, and porosity. Despite these differences, each mouse strain developed a mechanically competent bone at adulthood by coordinately adjusting multiple traits to maximize whole bone stiffness while minimizing mass. In addition to differences in external bone size, A/J and B6 femurs have been shown to differ at the gene expression level [14]. In our previous work, the gene showing the greatest expression level difference between the two strains was the *Wnt* antagonist *Sost*, which codes for sclerostin, a protein secreted by osteocytes that inhibits osteoblast differentiation by preventing the binding of *Wnt* ligand to LRP5/6 [1–4]. *Sost* was upregulated in A/J relative to B6 across growth, including a more than 2-fold difference at 6 weeks of age, that was translated to the protein level [14]. Further, a significant upregulation in *Sost* mRNA levels in A/J was seen across all time points during growth. Sclerostin protein levels were also significantly higher in the serum of A/J mice relative to B6 at 4 and 6 weeks of age (Schlecht et al. [14], unpublished data). It is unclear whether these differences in *Sost* gene expression or other differentially expressed genes within the *Wnt-*pathway contribute to the differences in external bone size between A/J and B6 mice. Nevertheless, these intrinsic differences in gene expression between A/J and B6 may have also contributed to the differential changes in whole bone strength following Scl-Ab administration. This interpretation is supported by the work of Morse and colleagues [34] that reported an anabolic response within the cortical matrix of sclerostin deficient mice with cyclic mechanical loading that translated to a significant increase in whole bone strength. Their work demonstrates that the relationship between sclerostin and whole bone function is complex with other genetic factors potentially playing an important role in establishing mechanical function. Similarly, Roschger et al. [35] found the anabolic effects following Scl-Ab treatment within the trabecula and cortex among transgenic mice with a severe OI phenotype do not translate to a substantial mechanical benefit in terms of max load and work. Again, this suggests the simple association between sclerostin and whole bone mechanical response is not straightforward and likely dependent on a suite of genetic factors within the *Wnt*-pathway. Given the nearly 2-fold greater change in whole bone strength of treated B6 femurs relative to treated A/J femurs, it will be important to begin teasing out the relative contributions of baseline geometry and differential gene expression profiles in future work.

Given the rather large changes in bone morphology observed in treated A/J and B6 mice, it was surprising to find few differences in gene expression levels between treated and control mice. We attribute this lack of differential gene expression to the study design, which limited RNA expression to a single time point at the end of treatment (8.5 weeks of age, 4 weeks after commencement of treatment). We chose the treatment timeframe of 4.5–8.5 weeks of age because it coincides with the time period in which mice begin to demonstrate adult ambulatory activity [36], and at a point we have previously shown *Sost* expression differences to be the most pronounced between A/J and B6 mice [14]. The fact that no difference in bone-related gene expression profiles was observed following treatment suggests that the gene expression differences driving the large changes in bone morphology occurred soon after treatment began then leveled off by the time the study was concluded or occurred in a cell type that was not included in our tissue lysates. As such, we do not anticipate that our RNA results were meaningful to understanding the response of these mice to Scl-Ab injections, and we chose not to follow up by confirming protein levels for *Camk2b or Fzd1*. A detailed time course study would be necessary to confirm our interpretation that lack of a differential gene expression response may reflect that long bones from both strains achieved a homeostatic state within 4 weeks of treatment initiation. Future studies should plan to assess gene expression differences earlier during the experimental treatment period to determine how the Scl-Ab treatment affected other.

In conclusion, A/J and B6 femurs showed different changes in whole bone strength following treatment with Scl-Ab during growth. Neutralization of sclerostin was associated with the expected large anabolic effect in both strains, but with the wide B6 femurs showing a greater change in whole bone strength compared to the narrow A/J femurs. The greater change intreated B6 femurs was attributed in part to the addition of tissue along the periosteal surface of a bone with wider baseline external size, consistent with engineering principles. The effect was further pronounced with tissue being added to both the periosteal and endocortical surfaces for A/J mice, which would be expected to limit the mechanical benefit. Known differences in gene expression profiles between A/J and B6 femurs likely contributed to this mechanical effect but the relative contributions of baseline geometry and differential gene expression profiles have yet to be determined. Our study is the first to report differential effects of Scl-Ab between two mouse strains that reflect normal variation in the skeletal traits of a human population. While targeted knockout experiments have contributed to our knowledge of the role that various genes play in bone biology, our study showed that there is also value to a systems-based approach to understanding the effects of perturbations such as Scl-Ab on the development of whole bone strength. While our use of Scl-Ab was as a perturbation to better understand bone development within our model system, our results may help lead to studies which assess the clinical results of Scl-Ab treatment.

## Supporting information

S1 Table Gene expression results for A/J-vehicle relative to B6-vehicle mice. (XLSM)

S2 Table Gene expression results for A/J-treated mice relative to A/J-vehicle mice. (XLSM)

S3 Table Gene expression results for B6-treated mice relative to B6-vehicle mice. (XLSM)
